# Pulsatilla Decoction Can Treat the Dampness-Heat Diarrhea Rat Model by Regulating Glycerinphospholipid Metabolism Based Lipidomics Approach

**DOI:** 10.3389/fphar.2020.00197

**Published:** 2020-03-03

**Authors:** Yong-li Hua, Qi Ma, Xiao-song Zhang, Ya-qian Jia, Xiao-ting Peng, Wan-ling Yao, Peng Ji, Jun-jie Hu, Yan-ming Wei

**Affiliations:** ^1^College of Veterinary Medicine, Gansu Agricultural University, Lanzhou, China; ^2^Institute of Animal Science, Southwestern University, Chongqing, China

**Keywords:** pulsatilla decoction, dampness-heat diarrhea, colon lipidomics, glycerophospholipid metabolism, arachidonic acid metabolism

## Abstract

**Ethnopharmacological Relevance:**

Diarrhea is a major medical problem in clinical practice. According to the theory of traditional Chinese medicine (TCM), different types of diarrhea should be treated with different TCM formulations based on the targeted medical condition. Dampness-heat diarrhea (DHD) is a serious diarrheal disease and Pulsatilla decoction (PD), a TCM, has been found effective against DHD.

**Objective:**

The aim of this study was to clarify the mechanism of action of PD in DHD using an untargeted lipidomics strategy.

**Materials and Methods:**

Wistar rats were randomized to four groups, including the control group, model group, PD groups and self-healing group. The PD groups were given a daily intragastric gavage of PD at doses of 3.76 g/kg. The rat model of DHD established by such complex factors as high-sugar and high-fat diet, improper diet, high temperature and humidity environment, drinking and intraperitoneal injection of Escherichia coli., which imitated the inducing conditions of DHD. Then the clinical symptoms and signs, blood routine, serum inflammatory cytokines levels and the histopathological changes of main organs were detected and observed to evaluate DHD model and therapeutic effect of PD. Lipid biomarkers of DHD were selected by comparing the control and model groups with the colon lipidomics technology and an ultra-high performance liquid chromatography (UHPLC) coupled with Q Exactive plus mass analyzer. Multivariate statistical analysis and pattern recognition were employed to examine different lipids within the colon of PD-treated rats.

**Results:**

The clinical symptoms and signs of the model rats were consistent with the diagnostic criteria of DHD. After treatment with PD, the clinical symptoms and signs of the rats with DHD were improved; the indexes of blood routine and inflammatory cytokines levels tended to be normal. The lipidomics profile of the model group were evidently disordered when compared to the control group. A total of 42 significantly altered lipids between the model-control groups were identified by multivariate statistical analysis. DHD may result from such lipid disorders which are related to glycerophospholipid metabolism, arachidonic acid (AA) metabolism, and sphingolipid metabolism. After PD treatment, the lipidomic profiles of the disorders tended to recover when compared to the model group. Twenty lipid molecules were identified and some glycerophospholipids and AA levels returned close to the normal level.

**Conclusion:**

Glycerophospholipid metabolism may play an important role in the treatment of dampness-heat induced diarrhea using PD.

## Introduction

Diarrhea is one of the most common diseases and serves as a public health issue with potentially fatal implications. Diarrhea is also a major health threat to the world’s population, both in subtropical and tropical developing countries (Hu Rui, 2012; Schiller, 2018). As diarrhea is a public health problem with potentially deadly effects, the WHO teamed up with other international organizations to launch a diarrheal disease control program with an emphasis on the use of traditional drug treatments to tackle the condition on a global scale (Xiong et al., 2018). Herbs and formulas in TCM can serve as drug discovery resources and valuable therapeutic strategies (Xu et al., 2015; Lv et al., 2017); however, according to the Chinese medicine theory, different types of diarrhea require different TCM formulas. Diarrhea can be divided into different forms such as dampness-heat, dampness-cold, spleen yang deficiency syndrome (Xiong et al., 2018) etc. DHD is one of the most common forms of diarrhea. This pathogenic form is developed by a collection of heat and wetness. For diarrhea due to wetness and heat, seasonal febrile diseases manifest as fever, headache, pantalgia, fatigue, distention of abdomen, diarrhea, oliguria with yellow urine, yellowish thick tongue coating, floating, and rapid pulse. The main clinical symptoms of DHD include pathological changes in the intestinal tract, intestinal mucosal injury and inflammation, as well as systemic inflammation (Zheng et al., 2016; Yao et al., 2017).

PD, a famous prescription for the treatment of DHD, was first recorded by Zhongjing Zhang in “Shanghan Lun (Treatise on Cold Pathogenic Diseases),” approximately 1800 years ago. PD showed good effects in treating DHD (Fan et al., 2019; Hua et al., 2019), The total efficacies of treatment were 90.00% using PD and the PD group was superior to the control group in improving defecation frequency and hematochezia degree. PD is comprised of four herbs, Bai Tou Weng (Pulsatillae radix (Bge.) Regel), Huangbai (Phellodendron chinense Schneid), Huanglian (Coptis chinensis Franch), and Qinpi (Fraxinus rhynchophylla Hance). A solid theoretical and experimental foundation exist for the use of PD to treat DHD (Zhang et al., 2014). *Pulsatilla chinensis* (Bunge) Regel, which is listed in the Chinese Pharmacopeia, has been used to remove heat, expel miasma, and cool the blood to stop diarrhea (Li et al., 2012). For thousands of years, the dried bark of *Phellodendron amurense* Rup. has been used as a TCM to remove damp heat and cure heat accumulation in the intestines and stomach, diarrhea, and other syndromes (Li et al., 2006; Yan et al., 2016). *Coptis chinensis* Franch is a common component of traditional Chinese herbal formulae used to relieve abdominal pain and diarrhea (Tjong et al., 2011); *Fraxinus rhynchophylla* Hance is commonly used to treat gout, arthritis, diarrhea, and bacillary dysentery in clinics and can remove wetness and heat (Fu et al., 2014); and Huanglian, Huangbai, and Qinpi synergistically enhance the Baitouweng effect of PD. Based on modern pharmacology research, PD can exert anti-fungal effects *in vitro* (Yang et al., 2017), inhibit inflammatory cytokines (Hu et al., 2009; Yang L. et al., 2018) and prevent damages to pathogenic factors by influencing endothelial glycolysis (Zhang et al., 2014). Based on acute and sub-chronic toxicity, Pulsatilla granules might be considered safe for veterinary use (Jia et al., 2017). Previous studies have also confirmed that the root of *Radix Pulsatilla* has potential therapeutic use against giardiasis (Li et al., 2012) and the possible prevention of infection (for example HBV) by specifically increasing SOD activity to minimize superoxide-mediated toxicity and increase superoxide release in the liver (Yao et al., 2004). Although *in vivo* and *in vitro* researches have shown that PD exerts important pharmacological effects, only few have explored its effect against DHD based on the Chinese Traditional Medicine theory. In this study, we explored the pharmacological effects of PD using a rat DHD model that was built in our previous study based on the Chinese Traditional Medicine theory (Yao et al., 2017). As demonstrated in our previous study, changes in lipid metabolism play an important role in the process of DHD as the central colon system contains large amounts of lipids. The study of lipid profiles regulated by PD in the process of DHD is therefore important to elucidate the therapeutic mechanism of PD.

Lipidomics is a type of high-throughput analysis technology based on systemic analysis of lipid composition and changes in expression within organisms used as research models. Lipidomics analysis can be efficiently used to study the family of lipids, changes in lipid molecules and their function in various biological processes, and elucidate the biological activity involved in the process as well as the mechanism employed. Recent lipidomics analysis has commonly used the liquid chromatography-mass spectrometry (LC-MS) technology, and is mainly divided into untargeted or non-targeted and targeted analysis (Lee and Yokomizo, 2018). Among them, the untargeted analysis model can detect various types of lipids in a sample without bias, and perform systematic analysis (Drotleff et al., 2018). Targeted analysis is mainly used to elucidate specific lipid molecules with great selectivity and specificity achieved in the quantitative analysis (Xu et al., 2018). Therefore, LC-MS is an important tool for the characterization of specific physiological and pathological traits (Giles et al., 2018; Kohno et al., 2018), and lipidomics based on UHPLC-MS has been widely applied to elucidate many biological events (Kohno et al., 2018; Righetti et al., 2018; Xu et al., 2018; Yang L. et al., 2018). As lipid metabolism plays an important role in the process of DHD, some lipid metabolites including LysoPC (P-16:0), LysoPC (P-18:0), LPA (16:0/0:0), LysoPC (22:0) etc. were found to exhibit changes in the process of DHD (Hua et al., 2019). Therefore, we used the UHPLC-based Orbitrap^TM^ mass spectrometry system to perform untargeted lipidomics analysis, and the LipidSearch^TM^ software (Thermo Scientific^TM^) to identify lipids and preprocessing data. LipidSearch^TM^ processing software can perform original data extraction, lipid and peak identification, peak alignment, and quantitative analysis of integration.

## Materials and Methods

### Instrument, Reagents, and Materials

Q-Exactive Plus mass spectrometer (Thermo Scientific); UHPLC Nexera LC-30, an ultra-performance liquid chromatography system (Shimadzu); Low temperature high-speed centrifuge (Eppendorf 5430R); Chromatographic column: Waters, Acquity UHPLC CSH C18, 1.7 μm, 2.1 × 100 mm column; acetonitrile (Thermo Fisher), isopropyl alcohol (Thermo Fisher), methyl alcohol (Thermo Fisher), and ammonium formate (Sigma-Aldrich, 70221) were employed in this study.

IL-1β, IL-6, IL-2, and TNF-α ELISA kits were obtained from Neobioscience Biotechnology Co., Ltd. (Shenzhen, China). We purchased Enterotoxigenic *Escherichia coli* O101 (bacteria number: CVCC231) from the Chinese Institute of Veterinary Drug Control.

### Preparation of PD

Table 1 shows the relevant information for PD. The mixture of *Pulsatillae radix* (Bge.) Regel, *Phellodendron chinense* Schneid, *Coptidis chinense* Franch and *Fraxinus rhynchophylla* Hance was soaked for approximately 40 min in 10 x (v/w) distilled water and boiled for 30 min, twice. The filtering medium was collected and concentrated to 1.00 g/mL (crude drugs).

**TABLE 1:** The medicinal formula of PD.

Latin name (Chinese name)	Medicinal parts	Place of production	Voucher numbers	Weight (g)	Authenticated by	Deposited place
*Pulsatilla chinensis (Bunge)* Regel	Root	Jilin	GSAUTCM-20170401	12 g	Prof. Yanming Wei	The herbarium center of Gansu Agriculture University
*Phellodendron chinense* Schneid	Bark	Sichuan	GSAUTCM-20170402	10 g	Prof. Yanming Wei	The herbarium center of Gansu Agriculture University
*Coptidis chinense* franch	Rhizome	Sichuan	GSAUTCM-20170403	6 g	Prof. Yanming Wei	The herbarium center of Gansu Agriculture University
*Fraxinus rhynchophylla* Hance	Bark	Hebei	GSAUTCM-20170404	12 g	Prof. Yanming Wei	The herbarium center of Gansu Agriculture University

The main components of PD were further analyzed by HPLC (Agilent Technologies, Santa Clara, CA, United States), with an Agilent 1260 HPLC system and UV detection system. The analytes were isolated using an Agilent ZORBAX SB C18 column (4.6 × 250 mm, 5 μm) at a column temperature of 30°C. The mobile phase comprised of A (acetonitrile) and B (0.1% H_3_PO_4_ acidified water). The detection wavelengths were set at 205 and 335 nm, and the absorption spectra of compounds were recorded between 205 and 340 nm.

### Experimental Animals

Thirty-two Wistar rats (weight, 180–220 g; age, 6–8 weeks; SCXK (Gan) 2015-0006) were obtained from the experimental Animal Center of Lanzhou University. Rats were housed at room temperature (22 ± 3°C) and constant humidity (55 ± 15%) under a natural light cycle in the laboratory. Food and tap water were provided *ad libitum*. All procedures in this study were strictly performed according to the Guidelines of the Animal Care and Use Committee of Gansu Agricultural University, and the local Animal Research Welfare Committee approved the study. Rats were randomly divided into four groups: model group, control group, PD group, and self-healing group.

### Experimental Process

Based on our previous study, the experiment comprised of four stages: stage 1, the high fat and high sugar stage (10 days) where rats in the model, self-healing, and PD groups were fed sufficient chow combined with an oral gavage of lard (4 mL/200 g weight) on alternate days; stage 2, the high temperature and high humidity environment stage (5 days) where white wine (56 degrees Red Star Erguotou, 2 mL/200 g weight) were given to rats in the model, self-healing, and PD groups by gavage every morning, and the rats placed in a high temperature (33°C ± 2°C) and humid (93% ± 2%) chamber for 8 h once daily; stage 3, the *Escherichia coli* injection stage (3 days) where rats in the model, self-healing, and PD groups were intraperitoneally injected with 1.06 × 109 CFU/mL *Escherichia coli* suspension (0.2 mL/200 g) twice at intervals for 24 h, followed by feeding in a natural environment for 1 day. Rats in the control group were fed standard diet in the natural conditions and intraperitoneally injected with an equal volume of normal saline for 18 days. Rats in the model, self-healing, and PD groups were also given honey water (30%) in all three stages (18 days). For the final (fourth) stage (the treatment stage), the PD crude drug (2 g/kg × 1.88 = 3.76 g/kg/day, the formula for the dose translation was as follows: human dose of crude herbs in clinic × 0.0188/200 × 1,000 × the multiple of clinical equivalency dose. The human dose of crude herbs in clinic is 40 g in the Table 1. The dose of the PD extracts was equivalent to that of crude herbs based on the TCM prescription and the multiple of clinical equivalency dose is 1.) was administered by gavage once per day for 5 consecutive days; rats in the control and self-healing groups were gavaged with a volume of normal saline. Rats in the model group were sacrificed at this stage.

On the 24th day, serum samples were collected by administering 1% pentobarbital anesthesia to rats for pain relief. Serum samples were used to analyze metabolite variations and the results were shown in reference (Hua et al., 2019). The ileum and colon were removed, quickly subpackaged, and immediately fixed in 10% neutral formalin. A section of the colon was also obtained and stored at −80°C for lipidomics analysis.

### Relevant Indicators for Observation and Further Determination

#### Clinical Symptoms and Signs

We observed the hair color, mental activity status, behavior response capacity, feed intake, excrement and urine color state, tongue coating, urine volume and weight, etc. of rats. Body weights were measured every day for the duration of the experiment (24 days).

#### Blood Routine and Inflammatory Cytokines

A fully automatic blood analyzer (Mindray BC-5300, Mindray Corporation, Shenzhen, China) was used to detect routine blood indicators, including the number of WBC, MO, LY, PCV, RBC, HGB, and PLT.

Serum levels of IL-1β, IL-2, IL-6, and TNF-α in rats were detected using their respective ELISA kits.

#### Histopathological Observation

A 10% neutral formalin solution was used to fix the ileum and colon samples for more than 15 days. Paraffin sections were developed, stained with hematoxylin and eosin (H&E), and observed using a light microscope.

### Colon Lipidomics Profiling With UHPLC Nexera LC-30A

#### Sample Pretreatment Method

A 200-μL volume of water was added to 30 mg of colon sample and vortexed for 5 s. Subsequently, 240 μL of precooling methanol was added and the mixture vortexed for 30 s, followed by the addition of 800 μL methyl tert-butyl ether (MTBE). Vortexing was again performed and the mixture was stored at room temperature for 20 min before centrifugation at 8 000 × *g* for 15 min at 4°C. The upper organic phase was removed and the mixture was blown with dry nitrogen. Before the analysis, 400 μL of isopropyl alcohol was added to the mixture which was vortexed for 60 s and centrifuged at 8 000 × *g* for 15 min at 4°C. The upper layer of the sample was used for subsequent analysis.

#### Instrumental Analysis and Data Preprocessing

The samples were separated using a UHPLC Nexera LC-30A system. The column was maintained at 45 °C and elution performed at a flow rate of 300 μL/min; the injection volume was 2 μL. The mobile phase comprised of A, 10 mM ammonium formate acetonitrile solution (acetonitrile:water = 6:4, v/v) and B, 10 mM ammonium formate acetonitrile isopropyl alcohol solution (acetonitrile:isopropanol = 1:9, v/v). Using the Waters Acquity UHPLC system with a CSH C18 column (2.1 × 100 mm, 1.7 μm), a linear gradient elution was performed as follows: 0–7 min, 30% B; 7–25 min, 30%–100% B, 25.1–30 min, 3% B. The autosampler was maintained at 10°C. To avoid the influence of instrumentation error, signal fluctuation was detected using random order, even during sample analysis. Sampling of the queue was performed every eight samples by setting one of the QC samples to monitor and evaluate the stability and reliability of the experimental data.

After UHPLC separation, samples were analyzed using a Q Exactive^TM^ plus mass spectrometer for mass spectrometry analysis using the following parameters: ion mode, positive; heater temperature, 300°C; sheath gas flow rate, 45 arb; aux gas flow rate, 15 arb; sweep gas flow rate, 1 arb; spray voltage, 3.0 kV; capillary temperature, 350°C; S-Lens RF Level, 50%; and MS1 scan range, 200–1800. For negative ion mode: heater temperature, 300°C; sheath gas flow rate, 45 arb; aux gas flow rate, 15 arb; sweep gas flow rate, 1 arb; spray voltage, 2.5 kV; capillary temperature, 350°C; S-Lens RF Level, 60%; and MS2 scan range, 250–1800.

According to the methods used which were each fully scanned (full scan), 10 pieces of map (MS2 scan, HCD) were collected, and the lipid molecules and quality of the lipid debris charge ratio were collected. The resolution of MS1 was 70,000 at m/z 200 while the resolution of MS2 was 17500 at m/z 200.

### Data Preprocessing

Using the LipidSearch^TM^ software version 4.1, peak recognition, lipid extraction (secondary appraisal), peak, peak alignment, quantitative processing, etc. were performed using the following parameters: precursor tolerance, 5 ppm and product ion threshold, 5%. Lipid molecules with a RSD >30% were deleted. LipidSearch^TM^ processing software can perform original data extraction, lipid and peak identification, peak alignment, and quantitative analysis of integration. LipidSearch^TM^ includes eight categories, 300 class types, and approximately 1.7 million types of lipid molecules in the MS2 and MS3 databases. Data from the Orbitrap^TM^ mass spectrometer can be used to generate high resolution, high quality, fine degree, parent ion, ion and neutral loss scanning identification algorithms, and perform reliable lipid qualitative analysis systematically (Supplementary Table S1).

The missing value in the group with >50% of lipid molecules was deleted and the abundance values were obtained using total peak area normalization based on LipidSearch data extraction. Multivariate data analysis was performed using the software SIMCA-P 14.1 (Umetrics, Umea, Sweden) after the process of Pareto-scaling. Multi-dimensional statistical analysis including unsupervised main points analysis, PCA, least squares discriminant analysis supervision (PLS-DA), and orthogonal partial least squares discriminant analysis (OPLS-DA) were used to screen different lipids. Unidimensional statistical analysis was performed using the Student’s *t*-test, and multiple variation analysis and R software map were employed for the volcano, hierarchical cluster analysis, and correlation analysis.

### Statistical Analysis

The experimental data were expressed as mean ± S.D. ANOVA followed by the Duncan’s multiple range test was performed using SPSS16.0 (Chicago, United States). A *P*-value <0.05 was considered statistically significant.

## Results

### Identification of the Main Components in PD

**FIGURE 1:**
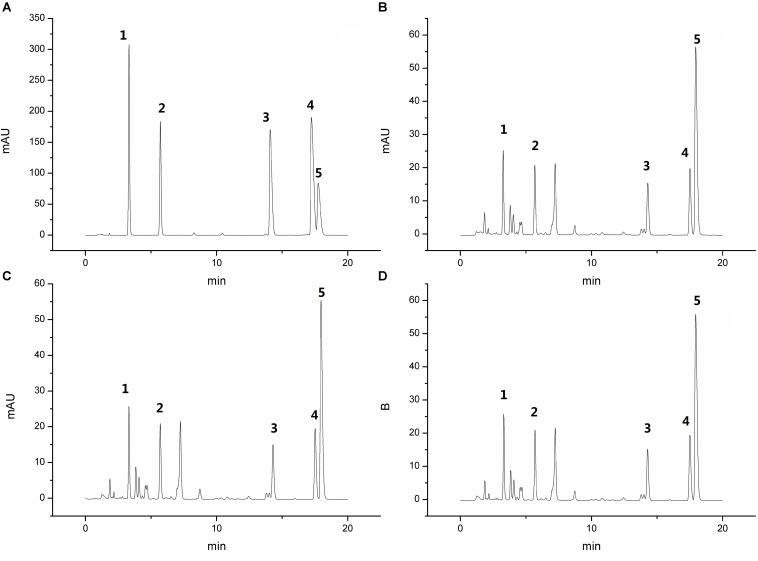
High performance liquid chromatography chromatogram of the standard mixture **(A)** and 3 PD samples **(B–D)**, Peaks 1: aesculin; 2: aesculetin; 3: jateorhizine hydrochloride; 4: palmatine chloride; and 5: berberine hydrochloride.

The HPLC chromatogram of the standard mixture and samples of PD is shown in Figure 1. All samples were significantly separated at the retention time of 20 min. Aesculin, esculetin, jateorhizine, palmatine, berberine, and anemoside B4 were quantitatively determined and the respective contents were 43.98, 31.13, 10.54, 15.31, 108.29, and 232.95 mg in 1 kg of the PD Crude herb.

### Clinical Symptoms and Signs

**FIGURE 2:**
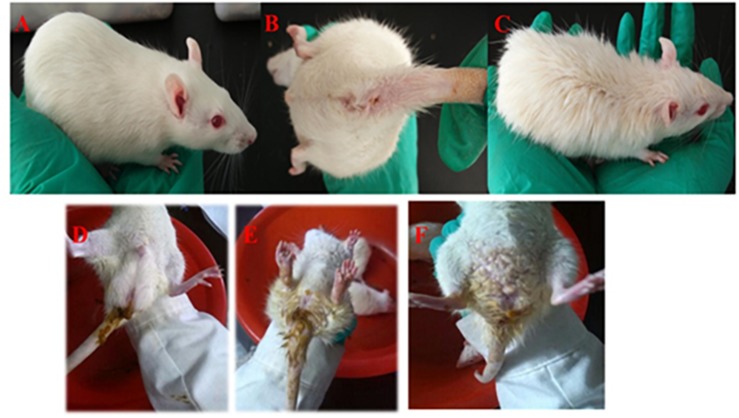
Symptoms of rats with DHD. Rats in the control group **(A,B)**. Rats treated in a high temperature and humidity environment, high-sugar and high-fat diet **(C)**. DHD symptoms such as sloppy diarrhea, anus redness, and swelling **(D–F)**, respectively.

Throughout the entire process of the experiment, rats in the control group did not display any clinical symptoms (23 days, Figure 2). At the first stage (10 days), rats in the three groups (model, self-healing, and PD groups) were fed a high-fat feed and honey sugar water. The excretory waste was characterized as soft and wet, and eventually became loose and appeared dry and yellow.

**FIGURE 3:**
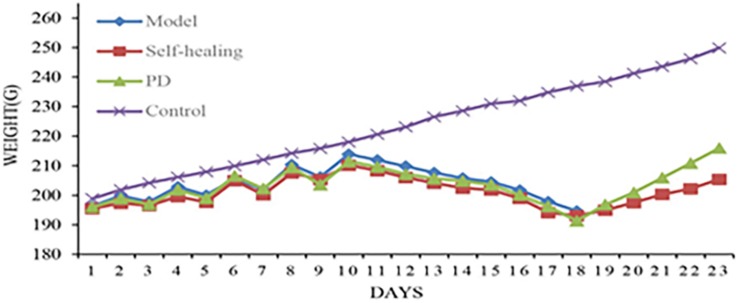
Body weight changes for rats in the control group, model group, self-healing group, and pulsatilla decoction treatment group during the experiment.

The waste collected from rats in the model, self-healing, and PD groups was soft and wet. In the days of fasting from water intake, urine output was increased and a pale-yellow color was apparent; urine during the days of feeding displayed high fat storage. The stool of rats went from soft and wet to loose, then finally appearing dry and yellow. Meanwhile, increased water intake, frequent urination and yellowish urine were observed, with a decrease observed on feeding days. The weight of rats increased on days of feeding and decreased on days of fasting. At the second stage (5 days), rats exhibited signs of depression and appetency, their hairs became lusterless, and stools appeared sticky and yellow, before becoming loose. In this stage, the weight of rats gradually decreased. At the third stage (3 days), rats in the model, self-healing, and PD groups displayed serious depression, sluggishness, chills, decreased food consumption, eyelid swelling and increased secretion, followed by diarrhea and increasing temperature. Meanwhile, stools were loose, sticky, yellow, pungent and overpowering; some stools also contained bloody mucus (Figure 2). Changes in weight were not significant over the 3 days; however, a gradual increase occurred in the subsequent experiments (Figure 3).

After PD administration (1 day), the spirit of rats began to recover, and a gradual increase in diet consumption and activity was observed. At 5 days following PD administration, symptoms were restored to normal and weights gradually increased.

These results demonstrated that the rat DHD model was successfully established, and the clinical symptoms and signs exhibited by the model rats could be improved by treatment with PD.

### Results of Blood Routine Analysis

Table 2 shows the increased levels of WBC, NE, MO, RBC, LY, HGB, and PCV in the self-healing and model groups compared to the control group (*P* < 0.05). These indexes for the PD treatment group were also lower than in the model group. Based on these results, strong inflammatory response and dehydration in the process of DHD were demonstrated, with PD possessing the ability to alleviate these symptoms.

**TABLE 2:** The effect of OVAS on blood physiology (mean ± SD).

	Control	Model	PD	Self-Healing
WBC (×10^9^/L)	10.79 ± 3.08	33.20 ± 4.29*	18.92 ± 5.53^Δ*^	20.19 ± 11.31^Δ*^
NE	30.85 ± 0.50	81.70 ± 3.78*	75.20 4.76^Δ*^	76.93 7.91^Δ*^
LY	59.50 ± 1.31	11.50 ± 3.67	15.83 ± 5.22	15.83 ± 7.93
MO	8.70 ± 0.85	4.02 ± 0.99	6.28 ± 2.31	5.60 ± 0.82
PCV	0.05 ± 0.71	1.30 ± 0.51	0.68 ± 0.42	0.47 ± 0.55
RBC (×10^12^/L)	6.38 ± 0.10	7.72 ± 2.00	6.51 ± 0.71	6.99 ± 1.32
HGB (g/L)	142.00 ± 2.83	165.50 ± 41.65	140.75 ± 14.42	145.33 ± 22.85
PLT (×10^9^/L)	975.00 ± 77.78	837.75 ± 243.33	579.50 ± 183.28	834.00 ± 175.32

White blood cell, WBC; red blood cell, RBC; hemoglobin, HGB; platelet count, PLT; number of neutrophils, NE; lymphocyte count, LY; mononuclear cells, MO; packed cell volume, PCV; *compared to the control group, significant increase, *P* < 0.05; Compared to the model, Δ represents significant decrease, *P* < 0.05.

### Results of the Changes in Inflammatory Cytokines

**FIGURE 4:**
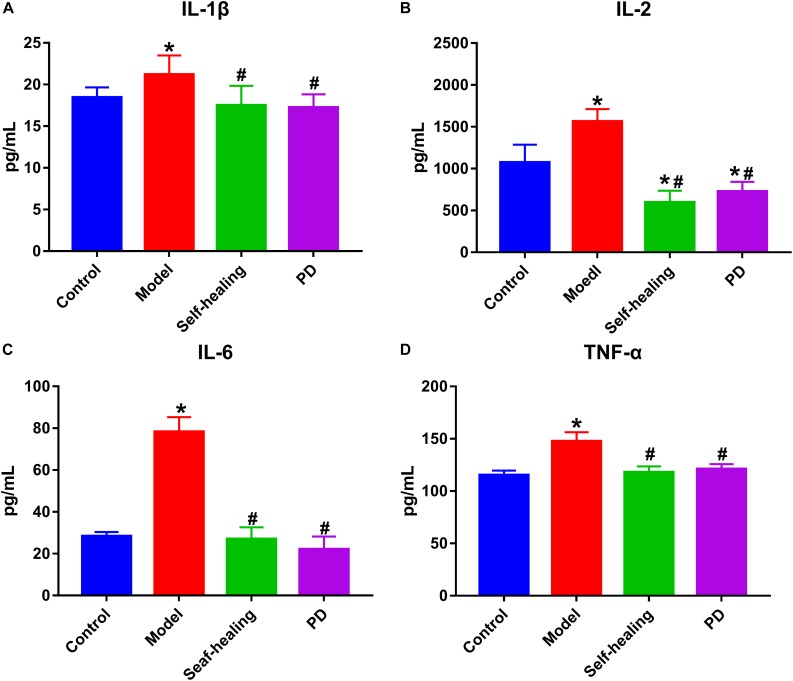
The contents of inflammatory cytokines in serum. **(A)** IL-1β, **(B)** IL-2, **(C)** IL-6, **(D)** TNF-α; control group; model group; Self-healing group; Pulsatilla decoction treatment group. *P < 0.05 vs control group; ^#^*P* < 0.05 vs model group.

The levels of TNF-α, IL-1β, IL-6, and IL-2 in the model group were increased compared to the control group (*P* < 0.05) (Figure 4), while the levels of these serum inflammatory cytokines decreased in the PD group when compared to the model group. These results are consistent with those obtained in blood routine detection.

### Histopathological Changes of Ileum and Colon

**FIGURE 5:**
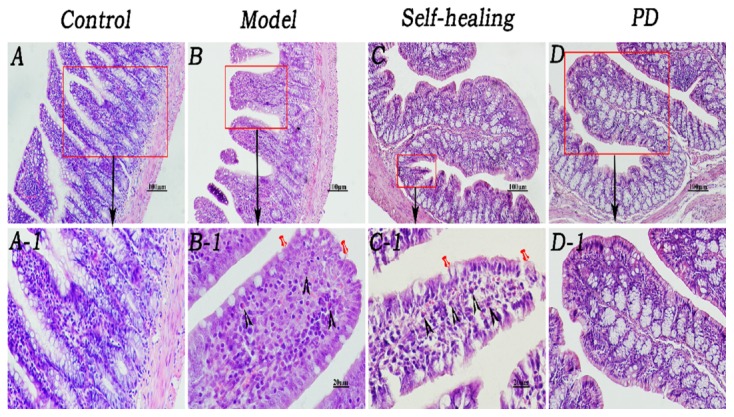
Histopathological changes of the ileum. **(A–A1)** Control group, the mucosal epithelium was integral, morphologies of the villi were normal, and venous congestion was not observed in the lamina propria (LP). **(B–B1)** Model group, infiltration of eosinophils was observed in the LP and around the crypt; integrity of the mucosal epithelium was destroyed and some epithelia were exfoliated; villi epithelium showed degeneration and necrosis. **(C–C1)** Self-healing group, eosinophils were reduced in the LP and around the crypt; integrity of the mucosal epithelium was destroyed, and parts of the epithelium were exfoliated; villi epithelium showed amelioration. **(D–D1)** PD group, epithelium was regenerated, integrity was recovered, and eosinophil infiltration decreased. Triangular arrows indicate eosinophil infiltration into LP. Pins indicate injuries of mucosal epithelium. Original magnification, × 400. Scale bar represents 20 μm.

**FIGURE 6:**
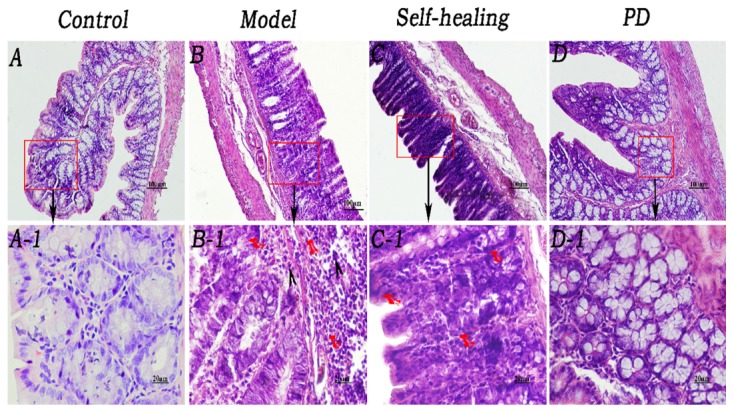
Histopathological changes of the colon. **(A–A1)** Control group, epithelium was integral, brush border was clearly visible, and morphologies of the veins in LP were normal although red cells could be seen within them. **(B–B1)** Model group, epithelium showed necrosis and exfoliation, and integrity was destroyed; few eosinophil infiltrations were present in the LP around the intestinal glands; many red cells were observed in the veins and arteries of LP. **(C–C1)** Self-healing group, epithelium showed necrosis and exfoliation and the integrity was destroyed; there was no eosinophil infiltration in the LP around the intestinal glands. **(D–D1)** PD group, mucosal epithelium was regenerated, integrity was recovered, neutrophil infiltration decreased in the LP, and congestion was effectively alleviated. Arrows in B and B1 indicate the injuries in the mucosal epithelium, few eosinophil infiltration and severe congestion, respectively. Original magnification, ×400. The scale bar represents 20 μm.

As shown in Figure 5, mucosal epithelium was complete and possessed a normal fuzzy morphology which cannot be observed in the LP venous congestion in the control group (Figures 5, 6A–A1). The integrity of the ileum and colon epithelium was disrupted, and inflammatory cellular infiltration and congestion were observed (Figures 5, 6B–B1) in the DHD rats and (Figures 5, 6C–C1) in the self-healing. In the PD group (Figures 5, 6D–D1), histopathological changes including the integrity of the ileum and colon epithelium, inflammatory cellular infiltration and congestion were effectively ameliorated or eliminated following treatment with PD.

### Results of Lipidomics

#### Comparison of the Base Peak Spectra of Samples

**FIGURE 7:**
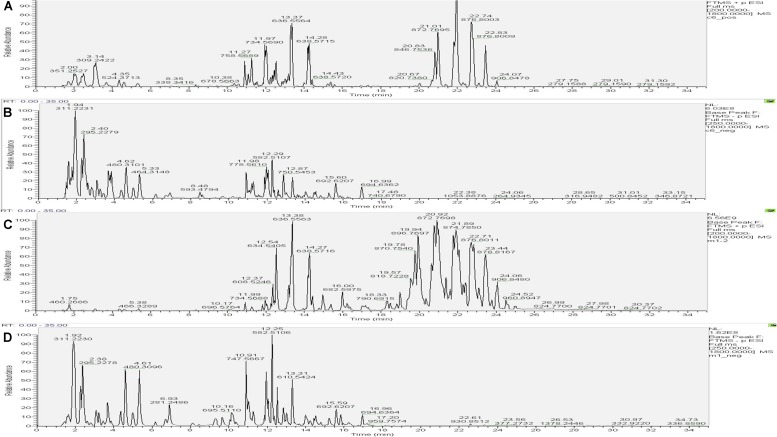
Typical base peak intensity chromatograms (BPC) for the colon of rats from the control group in positive ion mode **(A)** and negative ion mode **(B)**, and those from the model group in positive ion mode **(C)** and negative ion mode **(D)**.

**FIGURE 8:**
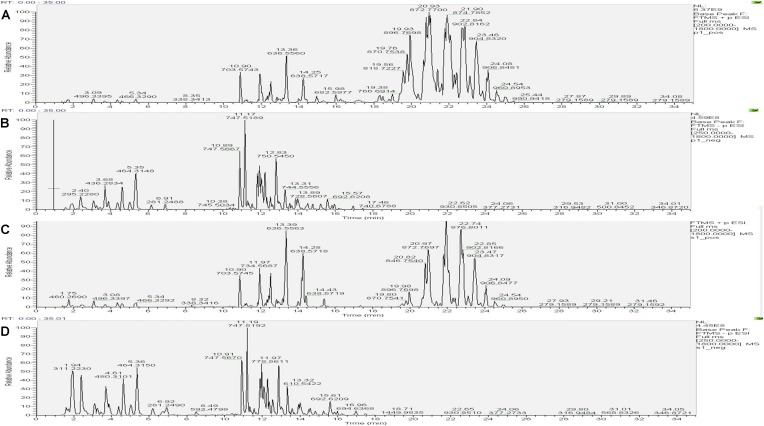
Typical base peak intensity chromatograms (BPC) for the colon of rats from the PD group in positive ion mode **(A)** and negative ion mode **(B)**, and those from the self-healing group in positive ion mode **(C)** and negative ion mode **(D)**.

The UHPLC-Orbitrap MS BPC of samples were compared in Figures 7, 8. The results showed an overlap between response strength and retention time of the chromatographic peak; the experimental repeatability was also deemed good.

#### Identification Number of Lipid Compounds

**FIGURE 9:**
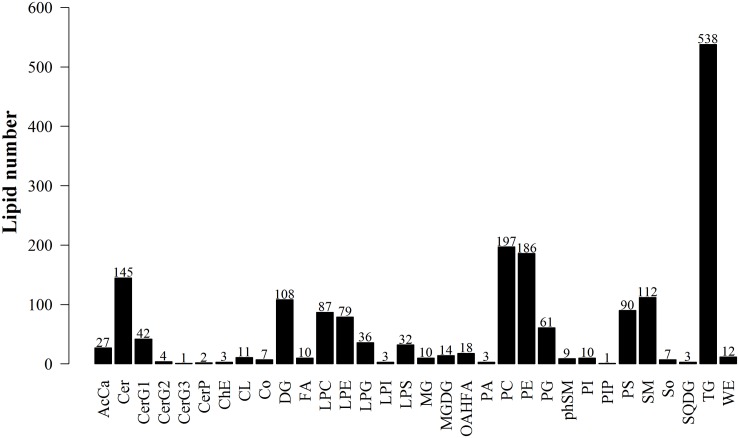
Lipid subgroup and lipid molecule count according to the International Lipid Classification and Nomenclature Committee and based on LipidSearchsoftware version 4.1.

The data obtained from negative and positive ion modes on the UHPLC-Orbitrap MS were analyzed to qualitatively identify then perform quantification using the Lipid Search software version 4.1. According to the International Lipid Classification and Nomenclature Committee, lipid compounds are divided into eight types. Each class type can be divided into different subtypes with polarity as the head of the class (lipid class). Each subgroup, based on differences that were not the saturation or length of the carbon chain, was divided into different molecular species (lipid species), to achieve a three-level classification of lipid compounds. This experiment in positive and negative ion modes identified 1868 lipid species and 32 lipid classes. Figure 9 shows the experimental identification based on the lipid classes, and all appraisal types to the number of lipid molecules.

#### Principal Component Analysis of the Total Samples

**FIGURE 10:**
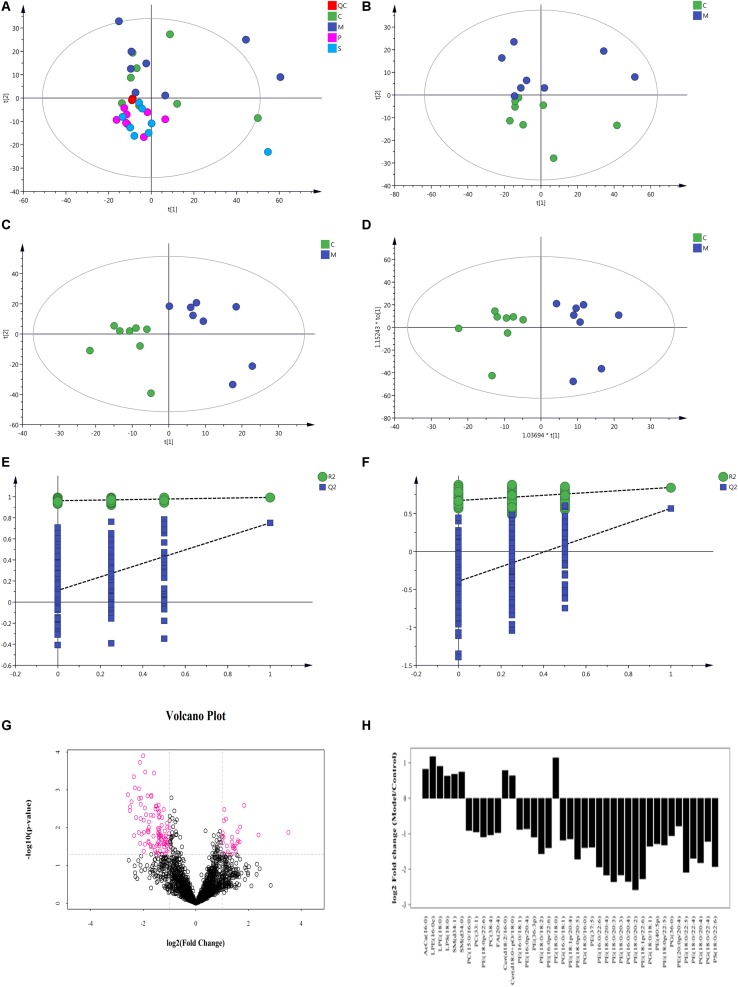
Multivariate data analysis of metabolites in rat colon based on UHPLC-Orbitrap MS. **(A)** PCA score plot of all samples and QC samples; **(B)** PCA score plot of Model-Control; **(C)** PLS-DA score plot of Model-Control; **(D)** OPLS-DA score plot of Model-Control; **(E)** PLS-DA displacement test of Model-Control; **(F)** OPLS-DA displacement test of Model-Control; **(G)** volcano plot of Model-Control, red point represents the different lipids (FC > 2.0, *P*-value < 0.05); **(H)** Fold-change analysis of the different lipids between Model and Control (C-Control, M-model, P-PD, S-self-healing).

After Pareto–scaling, the extraction peaks of all experimental and QC samples were analyzed by PCA. As shown in Figure 10A, the QC samples closely gathered and were found in the middle of all groups; this showed the repeatability of the objectives of the test. The PCA model parameter was obtained using a 7-fold cross-validation.

#### Lipid Molecule Multivariate Statistical Analysis

Non-supervised PCA was used to distinguish and survey changes in lipids in the model and control groups. Figure 10B shows the samples of the two groups in the PCA model; the two groups of samples on the PC1 and PC2 dimension charts exhibited a specific trend of separation. Subsequently, to show lipid distinctions and trends between the control and model groups, the PLS-DA model was established. Samples of the model group and control group were significantly separated, indicating that lipid metabolism was significantly changed due to diarrhea (Figure 10C). Both the R2Y and Q2 of this PLS-DA model were above 0.6 (Figure 10E), indicating good reliability. The different lipids were selected based on the variables, with VIP > 1.0 in the OPLS-DA model between the control and model groups (score plots, Figure 10D; response permutation testing of the model, Figure 10F). FC > 2.0 metabolites in the control and model groups were show in Figures 10G,H. A univariate statistical analysis was also used to verify whether lipids had significant difference. VIP > 1 and *P* < 0.05 were considered as significant differences for lipids. Forty-two lipids were identified, including 3 Sphingolipids, 2 Fatty Acyls, and 37 Glycerophospholipids. Table 3 shows the details of the 42 lipids. As shown in Figures 10E,F and Table 3, compared to the control group, the levels of glycerophospholipids including PE, PG, PS, and PC, except PE (18:0/18:0), were decreased in the model groups. In contrast, the levels of all SMs including SM and CerG (Simple Glc series), some glycerophospholipids such as LPE and LPS, and Fatty Acyls including AcCa (Acyl Carnitine) and FA were increased in the model groups.

**TABLE 3:** Differences in lipids between Model and Control group.

No.	Lipid ion	Ion formula	CalMz	RT (min)	Fold change	*P*-value	OPLS-DA VIP	Change trend	Statistical significance
43POS	AcCa(16:0)+H	C23 H46 O4 N1	400.3421355	3.04	1.77	0.046	1.09	↑	*
54POS	LPE(16:0e)+H	C21 H47 O6 N1 P1	440.3135535	3.8	2.26	0.030	1.30	↑	*
84POS	LPE(18:0)+H	C23 H49 O7 N1 P1	482.3241185	4.60	1.87	0.024	3.38	↑	*
143POS	LPS(18:0)+H	C24 H49 O9 N1 P1	526.3139485	3.45	1.55	0.047	1.11	↑	*
499POS	SM(d34:1)+H	C39 H80 O6 N2 P1	703.5748525	11.17	1.60	0.016	1.75	↑	*
507POS	SM(d34:0)+H	C39 H82 O6 N2 P1	705.5905025	11.31	1.67	0.049	1.48	↑	*
543POS	PC(15:0/16:0)+H	C39 H79 O8 N1 P1	720.5537835	11.45	0.53	0.017	1.82	↓	*
625POS	PC(33:1)+H	C41 H81 O8 N1 P1	746.5694335	13.32	0.52	0.027	1.57	↓	*
735POS	PE(18:0p/22:6)+H	C45 H79 O7 N1 P1	776.5588685	12.53	0.47	0.010	1.08	↓	*
848POS	PC(38:4)+H	C46 H85 O8 N1 P1	810.6007335	12.08	0.49	0.036	1.91	↓	*
1363NEG	FA(20:4)-H	O2 H31 C20	303.2329535	5.27	0.51	0.023	1.25	↓	*
1460NEG	Cer(d18:2/16:0)+HCOO	C35 H66 O5 N1	580.4946475	11.33	1.72	0.021	3.23	↑	*
1490NEG	Cer(d18:0+pO/18:0)+HCOO	C37 H74 O6 N1	628.5521625	12.72	1.55	0.027	1.43	↑	*
1554NEG	PE(16:0/18:1)-H	C39 H75 O8 N1 P1	716.5235805	12.33	0.54	0.035	2.59	↓	*
1562NEG	PE(16:0p/20:4)-H	C41 H73 O7 N1 P1	722.5130155	11.87	0.55	0.047	7.24	↓	*
1567NEG	PE(36:3p)-H	C41 H75 O7 N1 P1	724.5286655	12.02	0.47	0.007	2.14	↓	**
1598NEG	PE(18:0/18:2)-H	C41 H77 O8 N1 P1	742.5392305	12.52	0.34	0.040	2.91	↓	*
1605NEG	PE(16:0p/22:6)-H	C43 H73 O7 N1 P1	746.5130155	11.56	0.38	0.012	3.01	↓	*
1607NEG	PE(18:0/18:0)-H	C41 H81 O8 N1 P1	746.5705305	13.67	2.21	0.006	1.16	↑	**
1610NEG	PG(16:0/18:1)-H	C40 H76 O10 N0 P1	747.5181615	11.56	0.44	0.024	2.07	↓	*
1613NEG	PE(18:1p/20:4)-H	C43 H75 O7 N1 P1	748.5286655	11.93	0.45	0.017	3.70	↓	*
1615NEG	PE(18:0p/20:5)-H	C43 H75 O7 N1 P1	748.5286655	12.21	0.30	0.030	2.23	↓	*
1617NEG	PG(18:0/16:0)-H	C40 H78 O10 N0 P1	749.5338115	11.93	0.38	0.004	3.67	↓	**
1619NEG	PE(37:5)-H	C42 H73 O8 N1 P1	750.5079305	10.69	0.38	0.005	1.31	↓	**
1644NEG	PE(16:0/22:6)-H	C43 H73 O8 N1 P1	762.5079305	11.11	0.26	0.003	1.05	↓	**
1653NEG	PE(18:0/20:4)-H	C43 H77 O8 N1 P1	766.5392305	12.36	0.22	0.001	5.59	↓	**
1656NEG	PE(18:0/20:3)-H	C43 H79 O8 N1 P1	768.5548805	12.55	0.19	0.000	2.25	↓	**
1657NEG	PE(18:0/20:3)-H	C43 H79 O8 N1 P1	768.5548805	12.78	0.22	0.001	1.32	↓	**
1659NEG	PG(16:0/20:4)-H	C42 H74 O10 N0 P1	769.5025115	10.21	0.20	0.025	1.50	↓	*
1660NEG	PE(18:0/20:2)-H	C43 H81 O8 N1 P1	770.5705305	13.37	0.17	0.001	1.01	↓	**
1663NEG	PE(18:1p/22:6)-H	C45 H75 O7 N1 P1	772.5286655	11.64	0.21	0.005	1.27	↓	**
1673NEG	PG(18:0/18:1)-H	C42 H80 O10 N0 P1	775.5494615	12.47	0.39	0.016	2.70	↓	*
1677NEG	PE(40:5p)-H	C45 H79 O7 N1 P1	776.5599655	12.66	0.41	0.005	2.14	↓	**
1679NEG	PE(18:0p/22:5)-H	C45 H79 O7 N1 P1	776.5599655	13.22	0.40	0.017	1.51	↓	*
1681NEG	PG(36:0)-H	C42 H82 O10 N0 P1	777.5651115	12.67	0.48	0.027	1.38	↓	*
1684NEG	PE(20:0p/20:4)-H	C45 H81 O7 N1 P1	778.5756155	13.89	0.58	0.039	1.33	↓	*
1714NEG	PE(18:0/22:5)-H	C45 H79 O8 N1 P1	792.5548805	12.73	0.23	0.001	1.14	↓	**
1720NEG	PE(18:0/22:4)-H	C45 H81 O8 N1 P1	794.5705305	13.05	0.31	0.001	2.374	↓	**
1724NEG	PG(18:0/20:4)-H	C44 H78 O10 N0 P1	797.5338115	11.22	0.28	0.011	4.98	↓	*
1775NEG	PG(18:0/22:4)-H	C46 H82 O10 N0 P1	825.5651115	11.88	0.43	0.032	1.16	↓	*
1791NEG	PS(18:0/22:6)-H	C46 H77 O10 N1 P1	834.5290605	12.37	0.26	0.001	1.57	↓	**

“↑” stands for upgrading, “↓”stands for downgrading. *Stands for significant difference (*P* < 0.05). **Stands for very significant difference (*P* < 0.01).

**FIGURE 11:**
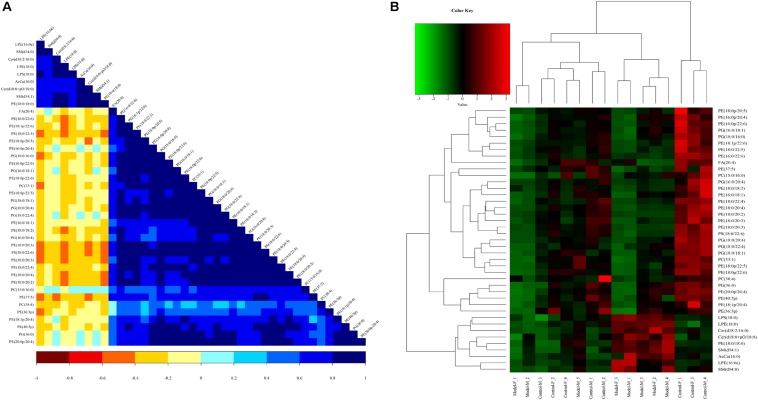
Heatmap visualization and hierarchical clustering analysis **(A)** and the correlation analysis results of the Control group **(B)** based on significant difference in lipids.

To assess the rationality of the differences between lipids and perform a comprehensive visualization to display the relationship between the samples and lipids for expression pattern differences in the different samples, each sample hierarchical clustering was analyzed using the expression quantity of the qualitative difference in lipids to assist in accurate lipid screening. As shown in Figure 11A, model and control groups can be divided into two large groups. The results of screening lipid differences were also proven to be correct.

A correlation analysis was used to evaluate the closely related degree among different lipids, and understand the changes in the biological state process. Figure 11B shows the correlation of different lipid molecules in the model-control. The same category of lipids can be clearly observed.

#### Results of PD Treatment in DHD Rats

**FIGURE 12:**
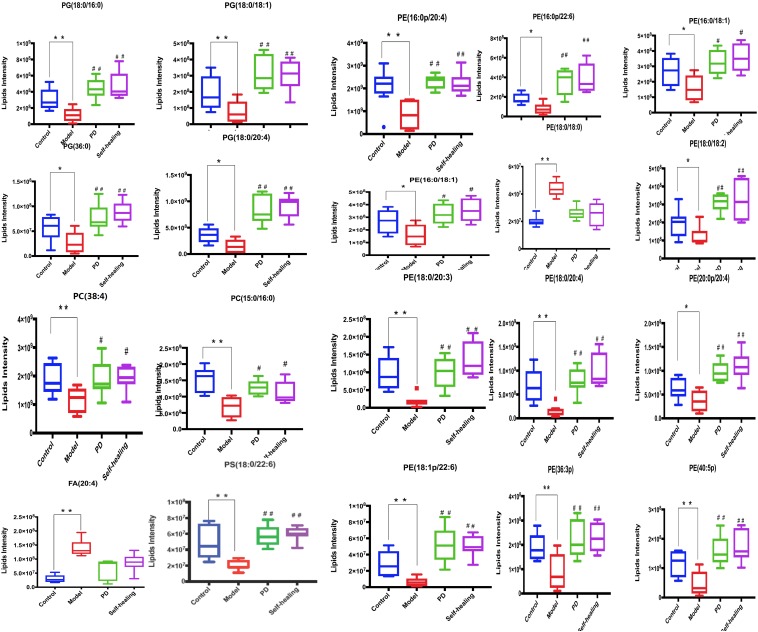
Differential expression levels (mean) of 20 differential lipids in different groups. A comparison of the relative intensities of the potential biomarkers in the control, model, PD, and self-healing groups. *P < 0.05 vs control group; ^#^*P* < 0.05 vs model group.

In the PD group, all lipids in the model-control showed a trend toward normal levels (Figure 12). Interestingly, the 19 differential lipids are glycerophospholipids, and 1 lipid, AA, is involved in AA metabolism. Analysis of the metabolic pathways indicated that the main lipids regulated by PD are involved in glycerophospholipid metabolism and AA metabolism.

**FIGURE 13:**
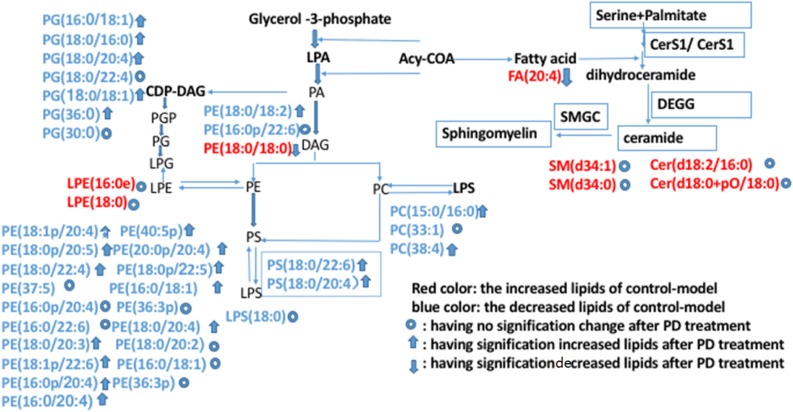
Possible metabolic pathway maps associated with the lipid biomarkers in dampness-heat induced diarrhea and dampness-heat induced diarrhea with PD treatment. Biomarkers in blue are those increased in the control-model. Biomarkers in red are those decreased biomarkers in the control-model. The up arrows represent biomarkers which were significantly increased after PD treatment. The down arrows represent the biomarkers which were significantly decreased after PD treatment. The circles represent biomarkers which were not significantly changed after PD treatment.

Further analysis could be performed using the lipid network (Figure 13), which indicated the interaction between differential lipids.

## Discussion

In this study, the lipid profiles within the colon of rats were characterized, and the effect of PD on DHD lipid composition was uncovered. The composition of lipid compounds in the control, model, self-healing, and PD groups was observed to vary dramatically. To our knowledge, this is the first lipidomic study of the administration of PD to rats with DHD to identify the lipid profile that could aid in the diagnosis of the disease or the prediction of its evolution, and explore the underlying pathologic mechanisms. One of the strengths of our study lies in glycerophospholipid metabolism and AA metabolism, which have an important role in the treatment of DHD with PD.

### Change of Glycerophospholipid Metabolism

Glycerophospholipid metabolism plays an important role in the DHD process. This is because diarrhea results from abnormalities in intestinal electrolyte transport (Singh et al., 2014; Das et al., 2018) which contributes to fluid and electrolyte homeostasis (Das et al., 2018). Intestinal ion transport mechanisms play a significant role in diarrhea (Sheikh et al., 2018). Therefore, the possible novel target for diarrhea therapy is the intestinal ion transporters. Some studies have reported that LPA, a form of phospholipids similar to growth factors, have been shown to prevent or restore gastrointestinal diseases, such as stomach ulcer and diarrhea in rats (Lin et al., 2010; Afroz et al., 2018). Diarrhea is caused by a decrease in the absorption of fluids and salts resulting from intestinal absorption and secretion disorders. Substantial absorption of sodium and water in the intestine is mediated by sodium/H + exchanger 3 (NHE3) found in the lumen of the intestinal cells. LPA is an effective stimulant of NHE3 and intestinal fluid absorption, which transmits signals through LPA5. As the regulation of LPA5 depends on its interaction with NHERF2, LPA may be useful in treating some diarrheal diseases (Lin et al., 2010). As LPA is a phospholipid acid (PA) formed by the activity of digesting PLA2, PA is a potential ingredient in the treatment of this gastrointestinal disease (Tanaka et al., 2012). The conversion of PA into diacylglycerol (DAG) by LPPs is the commitment step for the production of PC, PE, and PS. DAG is also converted into CDP-DAG, which is a precursor for PG, phosphatidylinositol (PI), and phosphoinositides (PIP, PIP2, PIP3) (Daum, 1999). In our results, the levels of PC, PE, PG, and PS were decreased in DHD rats compared to the levels in control rats. This is because of the decrease in production of LPA in the DHD process. LPA has been reported to ameliorate epithelial damage in chemical-induced colitis in rats (Lee et al., 2011). Through *in vivo* study, LPA has also been identified as a key regulator of epithelial cell proliferation and migration in the intestine. As loss of LPA impedes mucosal restitution of wounded areas in the colon, demon-FIG 9 LPA1 requires epithelial mucosal wound repair, reinforcing the clinical importance of LPA1 (Lee et al., 2013). PD also demonstrated the potential to increase the levels of these metabolites. Thus, treatment of DHD with PD has a relationship with intestinal ion transporter mechanisms. Owing to this, we believe PD can promote the production of LPA, which can inhibit intestinal chloride secretion in preclinical *in vitro* studies and in NHE3 upregulation of DRA expression (Singla et al., 2012), and contribute significantly to PD treatment of DHD.

Saturated PC is an essential lipid component of pulmonary surfactant (Li and Vance, 2008) and PC has antibacterial effects. A previous study showed that PD also has antibacterial effects (Yang Y. et al., 2018). Therefore, the interference of PD with PC may change the ability of the body to resist bacteria.

### Change of AA Metabolism

AA is an important component of the cell membrane, and possesses fluidity and flexibility. The four double bonds of AA contributes to its ability to easily oxidize, thereby producing excessive metabolites, which are of great significance to the normal function of the immune system, promotion of allergy and inflammation, response to inflammation, and appetite stimulation (Hanna and Hafez, 2018). In the present study, the decreased levels of AA, TNF-α, IL-1β, IL-6, and IL-2, and the pathological observations demonstrated that dampness-heat induced diarrhea is associated with inflammation. Indeed, inflammation plays an important role in DHD and this has been demonstrated (Yao et al., 2017; Schiller, 2018). Interestingly, some studies have proven that lipid metabolism disorder could increase the incidence of inflammatory bowel disease (Fujimoto et al., 2013; Paik et al., 2013). In addition, high-sugar and high-fat diet can induce AA metabolism disorder, and oxidative stress is the pathogenic link that bridges over-nutrition with inflammation via NF-kB activation. Activated NF-kB can trigger the generation of inflammatory molecules such as TNF- α and IL-6 (Kalivarathan et al., 2017). The levels of these molecules are also increased in high-sugar and high-fat diet-fed rats because of NF-kB activation. Heat stress can cause hemorheology disorder, and extensive microcirculatory obstruction. Microcirculatory obstruction may further result in local hypoxia and energy metabolism disturbance, as demonstrated by the severe inhibition of oxidative phosphorylation. Together with the stimulation of the biological factor of *Escherichia coli*, colonic epithelium was destroyed. Therefore, fat metabolism disorder and microcirculatory obstruction are the pathological bases of colon injury in DHD, and is caused by AA metabolism disturbance induced by high-sugar, high-fat diet, high temperature, and a highly damp environment.

Degradation of glycerophospholipids by PLA2 can cause AA release (Frisardi et al., 2011). Non-enzymatic and enzymatic oxidation of AA produce several lipid mediators (Yang L. et al., 2018), all of which are closely associated with the glycerophospholipid pathways involved in DHD; this suggests that an interplay among lipids occurs in the colon tissue (Kasuga et al., 2015). The results demonstrated that the level of AA was significantly increased in the model group, whereas a significant decrease in AA was observed when compared to the model group, with restoration to normal level observed in the PD group. These results indicate that PD may exhibit treatment effects in DHD by suppressing the production of AA. In addition, a relationship is demonstrated to exist with the increase in glycerophospholipids.

## Conclusion

In this study, a lipidomics strategy-based UHPLC-Q Exactive^TM^ Plus mass spectrometry method was applied to examine the lipid profiles of DHD rats following treatment with PD, and further elucidate the possible mechanism of PD. Based on the repeatability of the QC results, the method was deemed reliable. Results from multivariate analysis showed that PD can treat DHD, and were consistent with the results of the pathological and physiological assays. When the lipid profiles in the colon of the control and model rats were compared, significant changes in the level of 42 lipids were observed. After PD treatment, 20 lipids including 19 glycerophospholipid and AA appeared normal. PD can therefore treat DHD by regulating glycerophospholipid metabolism and AA metabolism.

## Ethics Statement

All procedures in this study were strictly performed according to the Guidelines of Animal Care and Use Committee of Gansu Agricultural University, and the local Animal Research Welfare Committee approved the study.

## Author Contributions

YH and QM conceived and designed the experiments and wrote the manuscript. XZ and WY performed the experiments. PJ and JH analyzed the data. YJ and XP performed the experiments. YW performed the analysis following constructive discussions. All authors read and approved the final version of the manuscript.

## Conflict of Interest

The authors declare that the research was conducted in the absence of any commercial or financial relationships that could be construed as a potential conflict of interest.

## Abbreviations

AA: arachidonic acid
AcCa: fatty acyls acyl carnitine
ANOVA: one-way analysis of variance
BPC: base peak spectra
CerG: simple glcserie
DHD: dampness-heat diarrhea
ELISA: enzyme linked immunosorbent assay
FA: fatty acid
HGB: hemoglobin concentration
HPLC: high performance liquid chromatography
IL: interleukin
LC – MS: liquid chromatography – mass spectrometry
LP: lamina propria
LPA: Lysophospholipids
LPE: lysophosphatidylethanolamine
LPS: lysophosphatidylserine
LY: lymphocytes
MO: mononuclear cells
OPLS-DA: orthogonal projection to latent structures–discriminant analysis
PC: phosphatidylcholine
PCA: principal component analysis
PCV: red blood cells deposited
PD: pulsatilla decoction
PE: phosphatidylethanolamine
PG: phosphatidylglycerol
PLA2: Phospholipase A2
PLT: the number of platelets
PS: phosphatidylserine
RBC: number of red blood cells
RSD: relative standard deviation
SM: sphingomyelin
TCM: traditional chinese medicine
TNF: tumor necrosis factor
UHPLC: ultra-high performance liquid chromatography
VIP: variable importance in the projection
WBC: white blood cell
WHO: World Health Organization.
